# Do food trichomes occur in *Pinguicula* (Lentibulariaceae) flowers?

**DOI:** 10.1093/aob/mcaa123

**Published:** 2020-06-27

**Authors:** Krzysztof Lustofin, Piotr Świątek, Piotr Stolarczyk, Vitor F O Miranda, Bartosz J Płachno

**Affiliations:** 1 Department of Plant Cytology and Embryology, Institute of Botany, Faculty of Biology, Jagiellonian University in Kraków, 9 Gronostajowa Street, 30-387 Cracow, Poland; 2 Institute of Biology, Biotechnology and Environmental Protection, Faculty of Natural Sciences, University of Silesia in Katowice, 9 Bankowa Street, 40-007 Katowice, Poland; 3 Unit of Botany and Plant Physiology, Institute of Plant Biology and Biotechnology, University of Agriculture in Kraków, 29 Listopada 54 Street, 31-425 Kraków, Poland; 4 Universidade Estadual Paulista (Unesp), Faculdade de Ciências Agrárias e Veterinárias, Jaboticabal, Departamento de Biologia Aplicada à Agropecuária, São Paulo, Brazil

**Keywords:** Butterworts, carnivorous plants, floral micro-morphology, food hairs, Lentibulariaceae, trichome structure, *Pinguicula*, spur, trichomes

## Abstract

**Background and Aims:**

Floral food bodies (including edible trichomes) are a form of floral reward for pollinators. This type of nutritive reward has been recorded in several angiosperm families: Annonaceae, Araceae, Calycanthaceae, Eupomatiaceae, Himantandraceae, Nymphaeaceae, Orchidaceae, Pandanaceae and Winteraceae. Although these bodies are very diverse in their structure, their cells contain food material: starch grains, protein bodies or lipid droplets. In *Pinguicula* flowers, there are numerous multicellular clavate trichomes. Previous authors have proposed that these trichomes in the *Pinguicula* flower play the role of ‘futterhaare’ (‘feeding hairs’) and are eaten by pollinators. The main aim of this study was to investigate whether the floral non-glandular trichomes of *Pinguicula* contain food reserves and thus are a reward for pollinators. The trichomes from the *Pinguicula* groups, which differ in their taxonomy (species from the subgenera: *Temnoceras*, *Pinguicula* and *Isoloba*) as well as the types of their pollinators (butterflies/flies and bees/hummingbirds), were examined. Thus, it was determined whether there are any connections between the occurrence of food trichomes and phylogeny position or pollination biology. Additionally, we determined the phylogenetic history of edible trichomes and pollinator evolution in the *Pinguicula* species.

**Methods:**

The species that were sampled were: *Pinguicula moctezumae*, *P. esseriana*, *P. moranensis*, *P. emarginata*, *P. rectifolia*, *P. mesophytica*, *P. hemiepiphytica*, *P. agnata*, *P. albida*, *P. ibarrae*, *P. martinezii*, *P. filifolia*, *P. gigantea*, *P. lusitanica*, *P. alpina* and *P. vulgaris*. Light microscopy, histochemistry, and scanning and transmission electron microscopy were used to address our aims with a phylogenetic perspective based on *matK/trnK* DNA sequences.

**Key Results:**

No accumulation of protein bodies or lipid droplets was recorded in the floral non-glandular trichomes of any of the analysed species. Starch grains occurred in the cells of the trichomes of the bee-/fly-pollinated species: *P. agnata*, *P. albida*, *P. ibarrae*, *P. martinezii*, *P. filifolia* and *P. gigantea*, but not in *P. alpina* or *P. vulgaris*. Moreover, starch grains were not recorded in the cells of the trichomes of the *Pinguicula* species that have long spurs, which are pollinated by Lepidoptera (*P. moctezumae*, *P. esseriana*, *P. moranensis*, *P. emarginata* and *P. rectifolia*) or birds (*P. mesophytica* and *P. hemiepihytica*), or in species with a small and whitish corolla that self-pollinate (*P. lusitanica*). The results on the occurrence of edible trichomes and pollinator syndromes were mapped onto a phylogenetic reconstruction of the genus.

**Conclusion:**

Floral non-glandular trichomes play the role of edible trichomes in some *Pinguicula* species (*P. agnata*, *P. albida*, *P. ibarrae*, *P. martinezii*, *P. filifolia* and *P. gigantea*), which are mainly classified as bee-pollinated species that had originated from Central and South America. It seems that in the *Pinguicula* that are pollinated by other pollinator groups (Lepidoptera and hummingbirds), the non-glandular trichomes in the flowers play a role other than that of a floral reward for their pollinators. Edible trichomes are symplesiomorphic for the *Pinguicula* species, and thus do not support a monophyletic group such as a synapomorphy. Nevertheless, edible trichomes are derived and are possibly a specialization for fly and bee pollinators by acting as a food reward for these visitors.

## INTRODUCTION

Plants offer various floral rewards for pollinators that can be divided into two groups: non-nutritive rewards (e.g. nest materials, a place of shelter, heat sources, substances for production of sexual attractants or places for mating ) and nutritive rewards (e.g. brood site, floral sweet tissue, stigmatic secretion or fatty oils) (Simpson and Neff, 1981). The most common floral nutritive rewards are nectar and pollen (Faegri and van der Pijl, 1979; Nicolson *et al.*, 2007). However, some species produce food bodies (including edible trichomes) that are eaten by their pollinators. The cells of these structures are rich with starch grains, protein bodies or oil droplets (Young, 1986; Thien *et al.*, 2009; for orchids, see Pansarin and Maciel, 2017 and references therein). Food bodies have been recorded in several unrelated plant families: Annonaceae, Araceae, Calycanthaceae, Eupomatiaceae, Himantandraceae, Orchidaceae, Pandanaceae, Nymphaeaceae and Winteraceae (e.g. Faegri and van der Pijl, 1979; Rickson, 1979; Cox, 1982; Young, 1986; Davies *et al.*, 2002; Thien *et al.*, 2009; Endress, 2010; Pansarin and Maciel, 2017). Thus, this type of reward occurs in both evolutionarily old families via beetle pollination (Annonaceae, Calycanthaceae, Eupomatiaceae, Himantandraceae, Nymphaeaceae and Winteraceae; see Endress, 2010) as well as in the more evolutionarily derived family Orchidaceae, which now represents an evolutionary pick of diversity. Floral food bodies can be divided into two major groups: the first (which occurs, for example, in the older lineages of angiosperms, Endress, 2010) – the outgrowths (or tips) of the carpels, stamens, staminodes and tepals; and the second – the epidermal edible trichomes. These trichomes have been particularly well analysed in Orchidaceae and they were found to have evolved independently in this family about five times (genera: *Cyanaeorchis*, *Dendrobium*, *Eria*, *Maxillaria* and *Polystachya*; Pansarin and Maciel, 2017). In orchids, they are very diverse in their structure and morphology as well as in the storage of nutritive material in their cells (e.g. Davies *et al.*, 2002; Davies and Turner, 2004; Pansarin and Maciel, 2017).

*Pinguicula* is a monophyletic genus within the Lentibulariaceae L. family (Jobson *et al.*, 2003; Müller *et al.*, 2004; Fleischmann and Roccia, 2018) and is among the Lamiales (Schäferhoff *et al.*, 2010; Chase *et al*., 2016) and contains about 96 species. *Pinguicula* are well known for their carnivory (e.g. Alcalá and Domínguez, 2003, 2005; Darnowski *et al.*, 2018; Heslop-Harrison, 1970; Heslop-Harrison and Heslop-Harrison, 1980; Vassilyev and Muravnik, 1988).

**Fig. 1.**
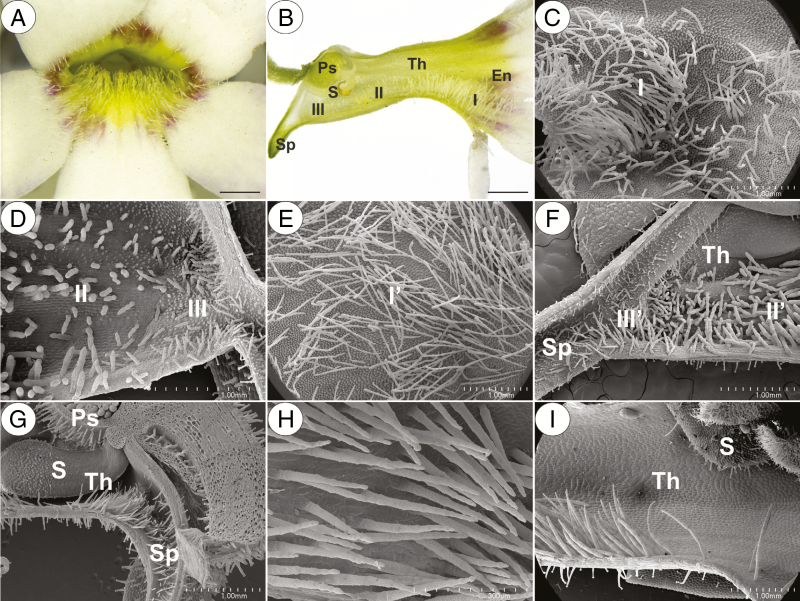
General morphology and micromorphology of the selected *Pinguicula* species that were examined. (A–D) General morphology and micromorphology of a *P. agnata* flower showing the entrance to the flower (En) with multicellular clavate slender trichomes (I), the throat (Th) with multicellular compact thick trichomes (II) in the front and two types of long and slender or short and compact non-glandular trichomes (III) that are located at the entrance to the spur (Sp); note the presence of a pistil (Ps) and a stamen (S) in the throat; scale bars = 2 mm, 2 mm, 1 mm and 1 mm, respectively. (E and F) Micromorphology of a *P. gigantea* flower; note the similar distribution and micromorphology of the non-glandular trichomes (Iʹ, IIʹ, IIIʹ) compared with *P. agnata*; scale bars = 1 mm and 1 mm, respectively. (G and H) Micromorphology of the *P. rectifolia* throat with generative organs and many celled uniseriate slender non-glandular trichomes indicated by an acute apical cell that is located in the throat and basal part of the spur; scale bars = 1 mm and 300 µm, respectively. (I) Micromorphology of the *P. hemiepiphytica* throat with long and slender multicellular non-glandular trichomes and a stamen; scale bar = 1 mm.

*Pinguicula* produce spurred zygomorphic flowers, which have nectar as a reward (Abrahamczyk *et al.*, 2017; Fleischmann and Roccia, 2018; Lustofin *et al.*, 2019). In *Pinguicula* flowers, there are numerous multicellular clavate trichomes at the base of the corolla – the throat; see Fig. 1A–I (Casper, 1966). Previous authors have proposed that these trichomes in the *Pinguicula* flower play the role of ‘futterhaare’ (‘feeding hairs’) and are eaten by their pollinators, or that some of them play the role of mimic pollen grains (see Fleischmann, 2016). Thus, the main aim of this study was to determine whether these trichomes of *Pinguicula* contain food reserves and thus may be a reward for potential pollinators. We selected species from the different clades, which are based on published phylogenetic proposals, within *Pinguicula* (members from three subgenera but focused on the Central American species) and also sampled species based on differences in their mating system. For this criterion, self- (i.e. a small flower with a whitish corolla) vs. outcross species (large, brightly coloured corollas, nectar guides and long spurs) were compared. Additionally, in our study, we considered the pollinator types (butterflies/fly and bees/hummingbirds). Fleischmann (2016) wrote that the clavate trichomes of *Pinguicula* are glandular, and therefore another task/aim was to determine whether these trichomes have the character of glands.

## MATERIALS AND METHODS

### Plant material

Seventeen taxa were sampled: *Pinguicula moctezumae* Zamudio & R.Z.Ortega, *P. esseriana* B.Kirchn., *P. moranensis* Kunth, *P. emarginata* Zamudio & Rzed., *P. rectifolia* Speta & F.Fuchs, *P. mesophytica* Zamudio, *P. hemiepiphytica* Zamudio & Rzed., *P. agnata* Casper, *P. albida* Wright ex Griseb., *P. ibarrae* Zamudio, *P. martinezii* Zamudio, *P. filifolia* C.Wright ex Griseb, *P. gigantea* Luhrs, *P. lusitanica* L., *P. alpina* L. and *P. vulgaris* L. [*P. vulgaris* subsp. *vulgaris* L. and *P. vulgaris* L. subsp. *bicolor* (Woł.) Á. Löve & D. Löve]. For our study, we primarily used living material (see Table 1). However, histochemical studies were used by some authors (e.g. Hernández and Katinas, 2019) in the case of herbarium material in order to show storage material or glandular structures. Therefore, we also used herbarium material of *Pinguicula* from the Herbarium of the Institute of Botany (KRA).

**Table 1.** List of the *Pinguicula* species that were examined along with information regarding their infrageneric classification, the origin of the plant material and the type of pollinator for each species.

Species	Infrageneric classification	Material origin	Type of pollinator
*P. moctezumae* Zamudio & R.Z.Ortega	*Temnoceras*	Botanical Garden of Jagiellonian University in Cracow (collected from: Mexico)	Lepidoptera (Abrahamczyk *et al.*, 2017)
*P. rectifolia* Speta & F.Fuchs	*Temnoceras*	Botanical Garden of Jagiellonian University in Cracow (collected from: Mexico)	Lepidoptera (flower’s structure indicates that type of pollinator)
*P. moranensis* Kunth	*Temnoceras*	Botanical Garden of Jagiellonian University in Cracow (collected from: near Santiago Juxtlahuaca, Oaxaca, Mexico 1851 m)	Lepidoptera (Villegas and Alcalá, 2018)
*P. emarginata* Zamudio & Rzed.	*Temnoceras*	Botanical Garden of Jagiellonian University in Cracow (collected from: Mexico)	Lepidoptera (flower’s structure indicates that type of pollinator)
*P. esseriana* B.Kirchn.	*Temnoceras*	Botanical Garden of Jagiellonian University in Cracow (collected from: Mexico)	Lepidoptera (flower’s structure indicates that type of pollinator)
*P. hemiepiphytica* Zamudio & Rzed	*Temnoceras*	Botanical Garden of Jagiellonian University in Cracow (collected from: near Ixtlan de Juarez, Oaxaca, Mexico, 2209–2535 m.)	Most probably hummingbirds (Lampard *et al.*, 2016)
*P. mesophytica* Zamudio	*Temnoceras*	Botanical Garden of Jagiellonian University in Cracow (collected from: Cerro Miramundo, El Salvador)	Ornithophily is presumed: a watercolour showing a species of hummingbird visiting a plants of *Pinguicula mesophytica* was shown in Roccia *et al.* (2016)
*P. agnata* Casper	*Temnoceras*	Botanical Garden of Jagiellonian University in Cracow (collected from: Mexico)	Diptera/Hymenoptera (flower’s structure indicates that type of pollinator)
*P. gigantea* Luhrs	*Temnoceras*	Botanical Garden of Jagiellonian University in Cracow (collected from: Mexico)	Diptera/Hymenoptera (Abrahamczyk *et al.*, 2017)
*P. ibarrae* Zamudio	*Temnoceras*	Botanical Garden in Liberec	Diptera/Hymenoptera (flower’s structure indicates that type of pollinator)
*P. martinezii* Zamudio	*Temnoceras*		Diptera/Hymenoptera (flower’s structure indicates that type of pollinator)
*P. albida* Wright ex Griseb.	*Temnoceras*		Hymenoptera (Dominguez *et al.*, 2014)
*P. filifolia* C.Wright ex Griseb.	*Temnoceras*		Hymenoptera (Dominguez *et al.*, 2014)
*P. lusitanica* L.	*Isoloba*	Botanical Garden of Jagiellonian University in Cracow (collected from: Europa)	Diptera/Hymenoptera(?), self-pollination (Heslop-Harrison, 2004)
*P. alpina* L.	*Pinguicula*	Herbarium of Jagiellonian University in Cracow (collected from: Alps, Innsbruck, Austria; KRA 0299930)	Diptera/Hymenoptera (Molau, 1993; Nordin, 2015)
*P. vulgaris* subsp.*vulgaris* L.	*Pinguicula*	Herbarium of Jagiellonian University in Cracow (collected from: Małe Pieniny, Rezerwat Zaskalskie, Poland; KRA 71415)	Diptera/Hymenoptera (Molau, 1993)
*P. vulgaris* L. subsp. *bicolor* (Woł.) Á. Löve & D. Löve	*Pinguicula*	Herbarium of Jagiellonian University in Cracow (collected from: Dąbrowa Górnicza, użytek ekologiczny ‘Młaki and Pogorią I’, Poland; KRA 0138573)	

### Methods

The flowers were examined using light microscopy (LM), scanning electron microscopy (SEM) and transmission electron microscopy as described below. The material was fixed in a mixture of 2.5 or 5 % glutaraldehyde with 2.5 % formaldehyde in a 0.05 m cacodylate buffer (Sigma; pH 7.2) overnight or for several days, washed three times in a 0.1 m sodium cacodylate buffer and post-fixed in a 1 % osmium tetroxide solution at room temperature for 1.5 h. Next, the material was treated as was previously described (Płachno *et al.*, 2017) and examined using a Hitachi H500 transmission electron microscope (Hitachi, Tokyo, Japan), which is housed at the University of Silesia in Katowice, at an accelerating voltage of 75 kV. The semi-thin sections (0.9–1.0 µm thick) that were prepared for LM were stained with aqueous methylene blue/azure II for 1–2 min (Humphrey and Pittman, 1974) and examined using Olympus BX60 and Nikon Eclipse E400 light microscopes to perform the general histology. The periodic acid–Schiff (PAS) reaction for LM (semi-thin sections) was also used to reveal the presence of insoluble polysaccharides (Wędzony, 1996), and Sudan Black B was used to detect the presence of lipids and cuticle material (Jensen, 1962).

Additionally, material that had been embedded in Technovit 7100 (Kulzer, Germany) was also examined. This material was fixed (as above), washed three times in a 0.1 m sodium cacodylate buffer, dehydrated in a graded ethanol series for 15 min at each concentration and kept overnight in absolute ethanol. Next, the samples were infiltrated for 1 h each in 3:1, 1:1 and 1:3 (v/v) mixtures of absolute ethanol and Technovit and then stored for 12 h in pure Technovit. The resin was polymerized by adding a hardener. The material was sectioned to 5 μm thickness using a rotary microtome (Microm, Adamas Instrumenten), stained with 0.1 % toluidine blue O and mounted in DPX (Sigma-Aldrich). The selected Technovit sections were stained with naphthol blue black (NBB) for total protein staining (Fisher, 1968; Mathe and Vieillescazes, 2002) or the PAS reaction was performed to visualize the starches (Wędzony, 1996).

In order to identify the main classes of the chemical compounds that are present in the trichomes, histochemical procedures with fresh or fixed flowers using Sudan III, Sudan Black B and Lugol’s solution were performed in order to detect the total lipids, starch grains and proteins (Johansen, 1940), respectively.

For SEM, the flowers were fixed (as above) and later dehydrated and critical point dried using CO_2_. They were then sputter-coated with gold and examined at an accelerating voltage of 20 kV using a Hitachi S-4700 scanning electron microscope, which is housed at the Institute of Geological Sciences, Jagiellonian University in Kraków, Poland.

### Phylogenetic analyses

The available *matK*/*trnK* DNA sequences of the *Pinguicula* species [*P. acuminata* (DQ010652.1), *P. agnata* (AF531782.1), *P. albida* (LC348432.1), *P. alpina* (AF531783.1), *P. ehlersiae* Speta & F.Fuchs (NC_023463.1), *P. elongata* Benj. (FM200224.1), *P. emarginata* (AF531785.1), *P. esseriana* (DQ010656.1), *P. filifolia* (AF531786.1), *P. gigantea* (AF531789.1), *P. gracilis* Zamudio (AF531790.1), *P. hemiepiphytica* (LC348445.1), *P. ibarrae* (LC348446.1), *P. laueana* Speta & F.Fuchs (DQ010659.1), *P. lusitanica* (DQ010661.1), *P. medusina* Zamudio & Studnička (LC348454.1), *P. moctezumae* (AF531797.1), *P. moranensis* (AF531798.1), *P. rectifolia* (AF531801.1), *P. rotundiflora* Studnička (AF531802.1), *P. sharpii* Casper & K.Kondo (AF531803.1) and *P. vulgaris* (AF531807.1)] were obtained from GenBank (NCBI) to be the ingroup. For the outgroup, two *Genlisea* [*G. aurea* A.St.-Hil. (NC_037078.1) and *G. violacea* A.St.-Hil. (NC_037083.1)] and two *Utricularia* species [*U. foliosa* L. (KY025562.1) and *U. reniformis* A.St.-Hil. (NC_029719.2)] were used. The sequences were aligned using the online MAFFT v. 7.450 package (Katoh *et al.*, 2019). All of the gaps were treated as missing. We used three approaches to create the phylogenetic reconstructions: Bayesian inference (BI), maximum likelihood (ML) and maximum parsimony (MP). BI was determined using Mr Bayes v. 3.2.7a (Ronquist *et al.*, 2012) under the CIPRES Science Gateway v. 3.3 (Miller *et al.*, 2010). For BI, 2 × 10^6^ generations were calculated using two runs with four chains until the standard deviation reached a value <0.01. In each run, the trees were sampled every 100 generations at a sample frequency of 100. The first 25 % of the trees that were initially produced were discarded as burn-in. The BI was conducted using the GTR + G model and was calculated using MrModeltest v. 2.4 software (Nylander, 2004) following the Akaike information criterion (Akaike, 1973). ML was determined using the online IQ-TREE v. 1.6.12 (Nguyen *et al.*, 2015) and the obtained branch supports with the ultrafast bootstrap (10 000 replicates) (Hoang *et al.*, 2018). For the MP analyses, PAUP* v. 4.0a (build 166) program (Swofford, 2002) was used under the CIPRES Science Gateway v. 3.3 (Miller *et al.*, 2010) to obtain the bootstrap values (2000 pseudoreplicates and a heuristic search with 1000 replicates with the random addition of sequences and the branch swapping algorithm TBR). The trees that were obtained were edited using FigTree v. 1.4.3 (Rambaut, 2016). To optimize the pollinators/syndromes on the tree, we used the BI tree, and the pollinators were plotted according to published studies (listed in Table 1). The pollinator silhouettes used in Fig. 4 were designed using Freepik (https://www.freepik.com).

## RESULTS

**Fig. 2.**
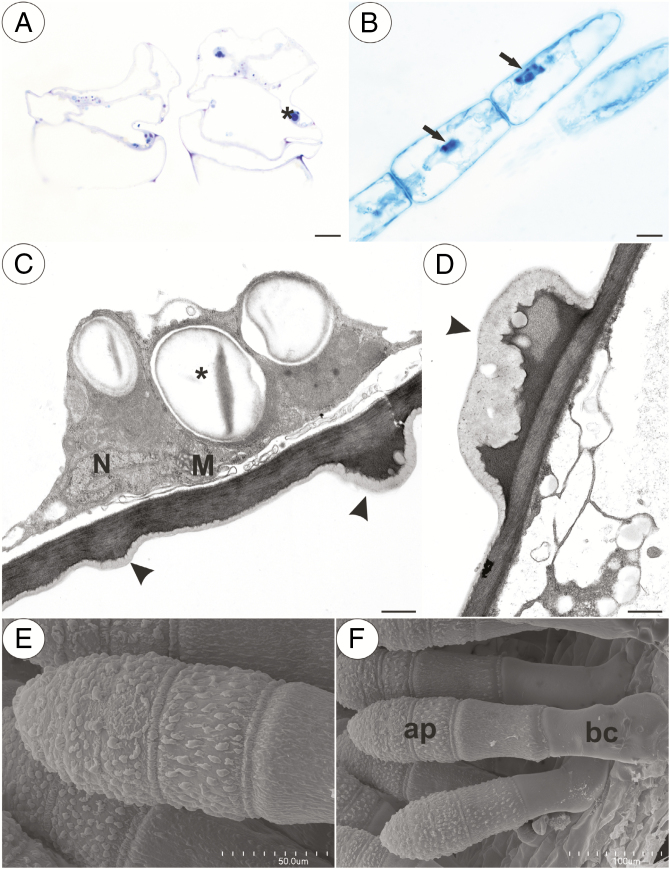
Structure of the non-glandular trichomes. (A) Section through the *P. albida* multicellular thick compact non-glandular trichomes that are located in the throat; note the numerous starch grains (asterisk); scale bar = 10 µm. (B) Naphthol blue black staining of a *P. moctezumae* multicellular non-glandular trichome showing the presence of a nucleus with a paracrystalline protein inclusion (arrow); note there are no protein bodies in the cytoplasm; scale bar = 10 µm. (C and D) Ultrastructure of a cell of a *P. agnata* non-glandular trichome; note the mitochondrion (M), nucleus (N) and prominent cuticular striations (arrowhead); scale bars = 0.7 µm and 0.5 µm, respectively. (E and F) Micromorphology of a *P. agnata* multicellular compact thick non-glandular trichome that is located in the front of the throat; note the cuticular striations on the surface of the apical cells (ap) and the smooth cuticle surface of the basal cell (bc); scale bars = 50 µm and 100 µm, respectively.

**Fig. 3.**
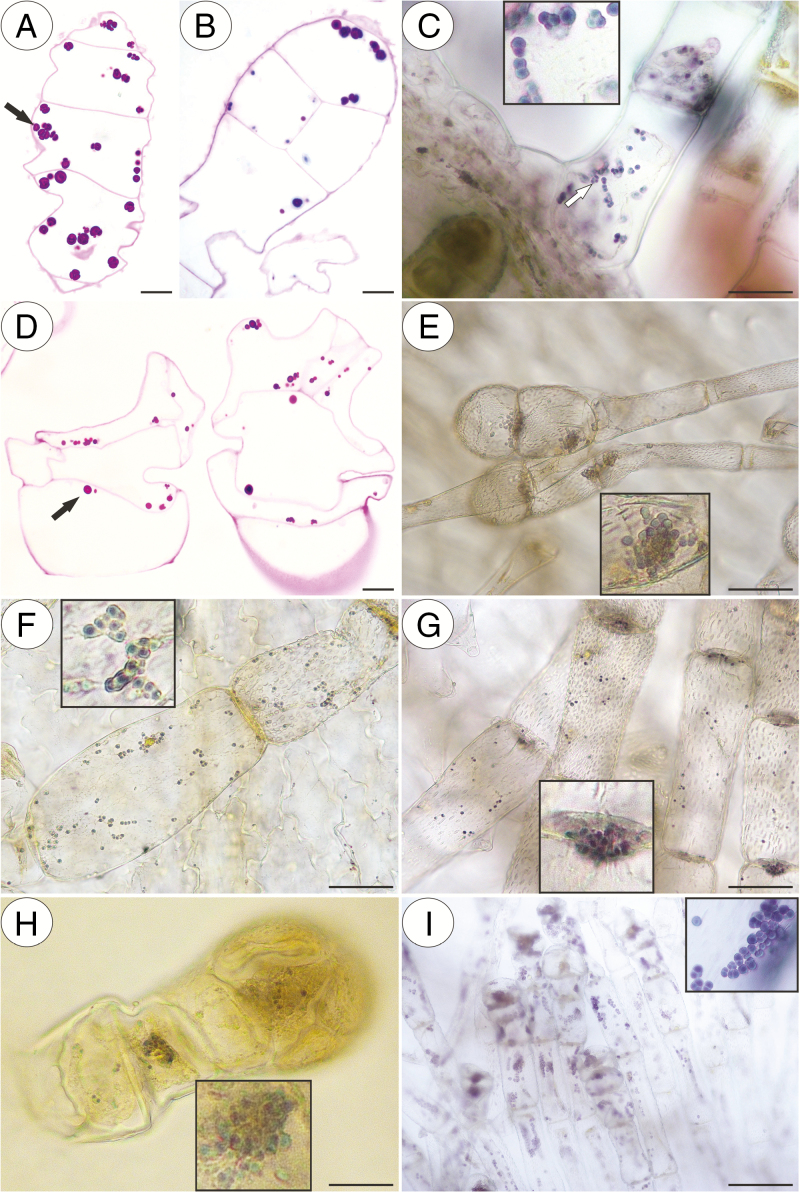
PAS reaction and Lugol’s staining of the *Pinguicula* species that were examined, which contain amyloplasts with starch grains (arrow, inserts) inside various types of non-glandular trichomes. (A–C) *P. agnata*; scale bars = 10 µm, 10 µm, 50 µm, respectively. (D and E) *P. albida*; scale bars = 10 µm and 50 µm, respectively. (F) *P. ibarrae*; scale bar = 50 µm. (G) *P. martinezii*; scale bar = 50 µm. (H) *P. filifolia*; scale bar = 50 µm. (I) *P. gigantea*; scale bar = 50 µm.

**Fig. 4.**
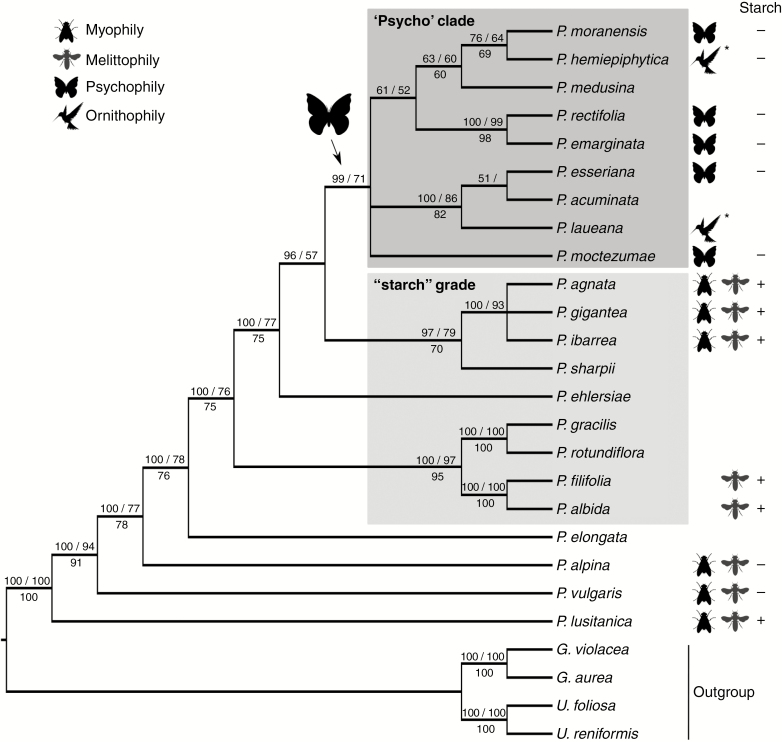
Phylogeny of the *Pinguicula* species based on the Bayesian inference (BI), maximum likelihood (ML) and maximum parsimony (MP) analyses of the *trnK/matK* sequences. The numbers above the branches refer to the BI posterior probability and the ML bootstrap support, respectively, and below the MP, the bootstrap support. The animal silhouettes denote the pollinator for each species. ‘*’ indicates the homoplastic origin of the ornithophily for *P. hemiepiphytica* and *P. laueana* independently. ‘+’ or ‘–’ indicate the presence/absence of starch grains in the edible trichomes of the bee-/fly-pollinated species.

**Fig. 5.**
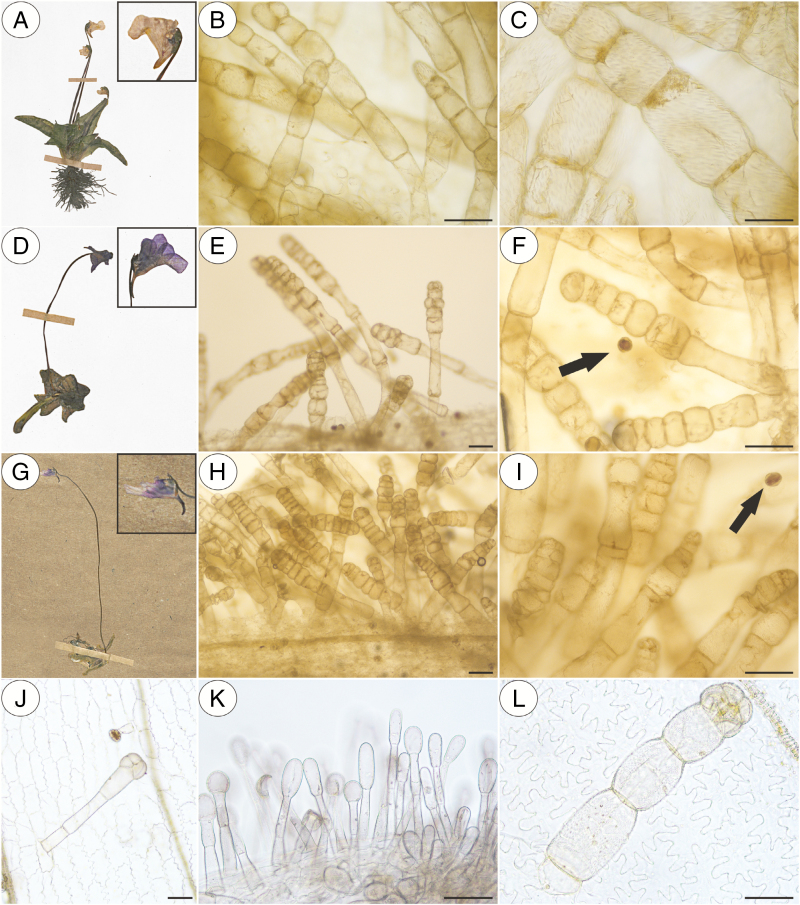
Histochemistry of the flower non-glandular trichomes from the species belonging to the *Pinguicula* and *Isoloba* subgenera; note the numerous starch grains inside the pollen grains (arrow). (A) Herbarium material of the *P. alpina* (KRA 0299930) that were examined. (B and C) Negative result of the Lugol’s staining of the *P. alpina* non-glandular trichomes; scale bars = 100 µm and 50 µm, respectively. (D) Herbarium material of the *P. vulgaris* subsp. *vulgaris* (KRA 71415) that were examined. (E and F) Negative result of the Lugol’s staining of the *P. vulgaris* subsp. *vulgaris* non-glandular trichomes; note the pollen grains (arrow) with a positive staining of the starch grains inside; scale bars = 100 µm and 100 µm, respectively. (G) Herbarium material of the *P. vulgaris* subsp. *bicolor* (KRA 0138573) that were examined. (H and I) Negative result of the Lugol’s staining of the *P. vulgaris* subsp. *bicolor* non-glandular trichomes; note the pollen grains (arrow) with a positive staining of the starch grains inside; scale bars = 100 µm and 100 µm, respectively. (J–L) Negative result of the Lugol’s staining of the *P. lusitanica* non-glandular trichomes; scale bars = 50 µm, 50 µm and 50 µm, respectively.

**Fig. 6.**
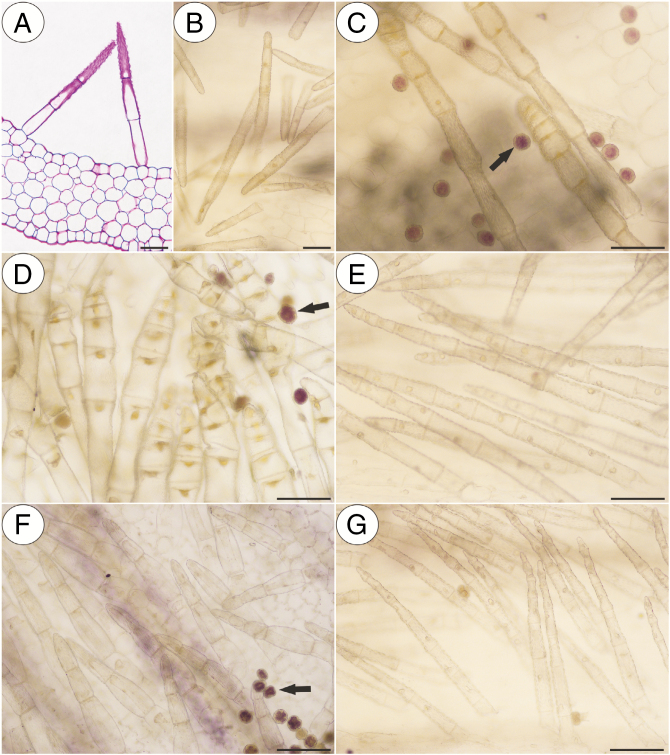
PAS reaction and Lugol’s staining of various non-glandular trichomes of the *Pinguicula* species that were examined that are pollinated by Lepidoptera; note the pollen grains (arrow) with a positive staining of the starch grains inside. (A) PAS reaction of the *P. moctezumae* non-glandular trichomes that are located in the basal part of the spur; scale bar = 50 µm. (B and C) Negative result of the Lugol’s staining of the *P. moctezumae* non-glandular trichomes; scale bars = 50 µm and 100 µm, respectively. (D) Negative result of the Lugol’s staining of the *P. esseriana* non-glandular trichomes; scale bar = 100 µm. (E) Negative result of the Lugol’s staining of the *P. moranensis* non-glandular trichomes; scale bar = 100 µm. (F) Negative result of the Lugol’s staining of the *P. emarginata* non-glandular trichomes; scale bar = 100 µm. (G) Negative result of the Lugol’s staining of the *P. rectifolia* non-glandular trichomes; scale bar = 100 µm.

**Fig. 7.**
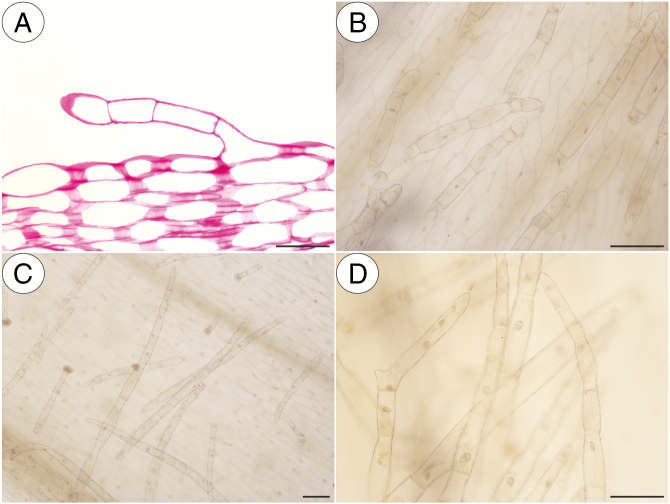
PAS reaction and Lugol’s staining of various non-glandular trichomes of the *Pinguicula* species that were examined that are most probably pollinated by hummingbirds. (A) PAS reaction of a *P. mesophytica* non-glandular trichome; scale bar = 50 µm. (B) Negative result of the Lugol’s staining of the *P. mesophytica* non-glandular trichomes; scale bar = 100 µm. (C and D) Negative result of the Lugol’s staining of the *P. hemiepiphytica* non-glandular trichomes; scale bars = 100 µm and 100 µm, respectively.

**Fig. 8.**
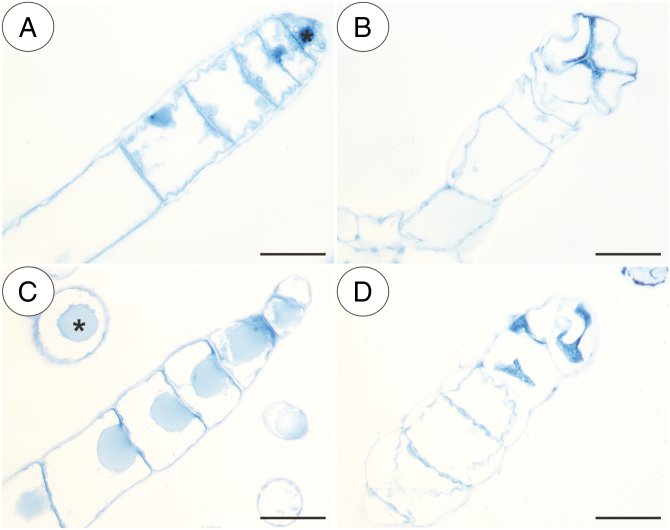
Naphthol blue black (NBB) staining of various non-glandular trichomes of the selected *Pinguicula* species that were examined; note the lack of protein bodies in the cytoplasm. Nucleus (asterisk). (A) *P. agnata*; scale bar = 50 µm. (B) *P. albida*; scale bar = 50 µm. (C) *P. esseriana*; scale bar = 50 µm. (D) *P. vulgaris*; scale bar = 50 µm.

**Fig. 9.**
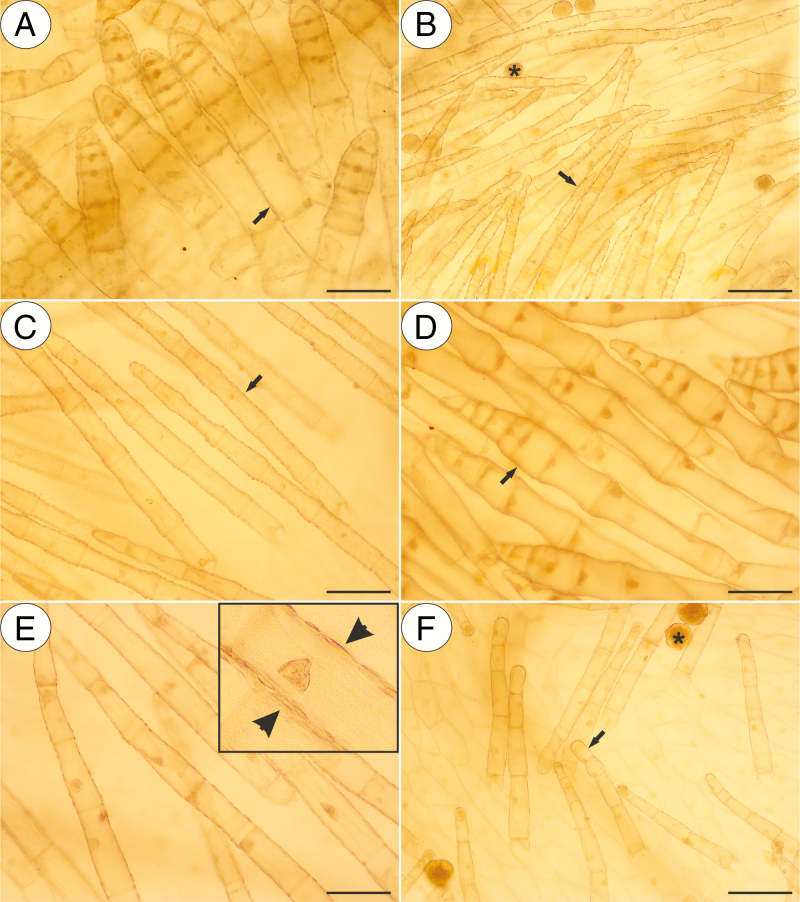
Sudan III staining of various non-glandular trichomes of the selected *Pinguicula* species that were examined; note the positive staining of the cuticular striations of the non-glandular trichomes cells (arrow, insert and arrowhead) and lipids inside the pollen grains (asterisk). (A) *P. agnata*; scale bar = 100 µm. (B) *P. rectifolia*; scale bar = 100 µm. (C) *P. moranensis*; scale bar = 100 µm. (D) *P. esseriana*; scale bar = 100 µm. (E) *P. hemiepiphytica*; scale bar = 100 µm. (F) *P. mesophytica*; scale bar = 100 µm.

In our study, we observed various types of multicellular non-glandular trichomes, which differed in terms of their micromorphology (see Supplementary data Table S1). The trichome cells were highly vacuolated (Fig. 2A, B) and contained a peripheral cytoplasm with organelles such as a nucleus, mitochondria, plastids and an endoplasmic reticulum (Fig. 2C). Intranuclear paracrystalline bodies occurred in the nuclei (Fig. 2B). Staining with NBB revealed that these consisted of proteins (Fig. 2B). Some trichome cells had visible cuticular striations (Fig. 2D–F), while others had a smooth surface (Fig. 2F). The PAS reaction and Lugol’s staining revealed amyloplasts with starch grains in the cells of the trichomes of the species from the subgenus *Temnoceras*: *P. agnata*, *P. albida*, *P. ibarrae*, *P. martinezii*, *P. filifolia* and *P. gigantea* (Fig. 3A–I and see ‘starch’ grade in Fig. 4). Starch grains were observed in these species independent of the type of trichomes (Supplementary data Table S1). Lugol’s staining did not reveal any amyloplasts with starch grains in the cells of the trichomes of the species from the subgenus *Pinguicula*: *P. alpina* (Fig. 5A–C) and *P. vulgaris* (*P. vulgaris* subsp. *vulgaris* and *P. vulgaris* subsp. *bicolor*) (Fig. 5D–I) or the subgenus *Isoloba*: *P. lusitanica* (Fig. 5J–L). Moreover, this staining did not reveal any amyloplasts with starch grains in the trichome cells of species from the subgenus *Temnoceras*, which is pollinated by butterflies [*P. moctezumae*, *P. esseriana*, *P. moranensis*, *P. emarginata* and *P. rectifolia*; Fig. 4 (‘psycho’ clade) Fig. 6A–G] or birds (*P. mesophytica* and *P. hemiepiphytica*, Fig. 7A–D; Supplementary data Table S1). Staining with NBB did not reveal any protein bodies (in either the cytoplasm or the vacuoles) in the cells of the trichomes of any of the examined species (Fig. 8A–D; Supplementary data Table S1). Staining with Sudan III did not reveal any lipid droplets in the cells of the trichomes in any of the examined species (Fig. 9A–F); however, positive staining was recorded in the cuticular striations (Fig. 9A–F).

The phylogenetic hypothesis, which was based on the *trnK/matK* sequences (Fig. 4), supports the assumption that both psychophily and ornithophily are derived for the *Pinguicula* lineages, probably from the plesiomorphic condition of myophily and/or melittophily. The ornithophily was possibly derived from the psychophily (Fig. 4). Thus, the pollination by birds has emerged at least twice as homoplasies to the *Pinguicula* species independently.

## DISCUSSION

We did not find the typical characters of glandular cells in the cells of the multicellular clavate trichomes. Therefore, we agree with Casper (1966, 2019) that these trichomes are non-glandular. We did show that the cells of the floral non-glandular trichomes of *P. agnata*, *P. albida*, *P. ibarrae*, *P. martinezii*, *P. filifolia* and *P. gigantea* were rich in amyloplasts that contained starch. Thus, these peculiar trichomes contain food reserves and probably function as edible trichomes. In orchids, edible trichomes (including pseudopollen-forming trichomes) are formed for a specific pollinator group, i.e. bees (Pansarin and Maciel, 2017). Thus, it is clear that in *Pinguicula* starch contained trichomes are recorded in species pollinated by bees, as showed in the ‘starch’ grade by the phylogenetical hypothesis (Fig. 4). Therefore, the lack of starch in the trichomes in the ‘psycho’ clade is a secondary loss, considering that *P. alpina*, *P. lusitanica* and *P. vulgaris* also did not present this character (Fig. 4). *Pinguicula mesophytica* is not represented in the tree but is a sister species to *P. moranensis* based on internal transcribed spacer (ITS) rDNA according to Shimai *et al.* (2007). Thus, pollination by birds is perhaps homoplastic in the *Pinguicula* species considering the known or supposed ornithophilic species (*P. hemiepiphytica*, *P. laueana* and *P. mesophytica*; Lampard *et al.*, 2016; Roccia *et al.*, 2016).

Interestingly, not all myophilic and melittophylic species had starch in these trichomes, which enabled us to infer that these traits are not a condition for those pollination syndromes. Moreover, we did not record food reserves in the trichomes of *P. alpina* and *P. vulgaris*, which are pollinated by bees and flies (Molau, 1993; Fleischmann, 2016). Fleischmann (2016) observed various dipterans dabbing at the yellow spots on the otherwise white corolla of *P. alpina* and on the white corolla marks on the violet corolla of *P. vulgaris* and *P. leptoceras* with their proboscis. He interpreted this behaviour as the insects trying to find nectar and pollen, and, therefore, in these species, the trichomes may guide insects to the spur. However, we do not agree with Fleischmann (2016) that they play the role of ‘feeding hairs’ in *P. alpina* and *P. vulgaris* because we did not find any reserve material in these trichomes. For this reason, these trichomes may play a tactile role and act as guides or they might mimic the edible trichomes of other species.

Most researchers accept that in *Pinguicula* the reward for pollinators is generally nectar because of the occurrence of a spur with glandular trichomes (Fleischmann and Roccia, 2018; Lustofin *et al.* 2019); however, actual observations of nectar secretion and nectar analysis are rare (Zamora, 1999; Abrahamczyk *et al.* 2017; Lustofin *et al.* 2019). Although edible trichomes may act as a reward in addition to nectar, a detailed study of nectar production and secretion in *Pinguicula* is required to be absolutely certain that all *Pinguicula* species produce nectar and in what quantities. In the related genera *Utricularia* (Hobbhahn *et al.*, 2006; Clivati *et al.*, 2014; Płachno *et al*., 2017, 2018, 2019*a*, *b*) and *Genlisea* (Aranguren *et al.* 2018), the reward for pollinators is nectar. However, in some species (*U. antennifera*, *U. capilliflora*, *U. dunlopii*, *U. dunstaniae* and *U. lowriei*), the spur is significantly reduced and the corolla forms filiform appendages (Taylor, 1989; Reut and Jobson, 2010). In *U. dunlopii*, the glandular trichomes (osmophores) are densely distributed on the modified floral appendages, and therefore their scent is most probably the attractant for visiting insects (Płachno *et al.*, 2016). Although there are yellow non-glandular trichomes in the flower throats of *U. multifida* and *U. tenella*, they do not play the role of edible trichomes (Płachno *et al.*, 2019*a*).

In orchids, the edible trichome cells (including the pseudopollen, which is formed by the disintegration of the trichomes) contain various types of food material (see Davies, 2009 and references therein). The main food material that is found in the edible trichome of orchids in the species from the *Maxillaria* genus is protein (Davies, 2009). Starch grains were recorded in the cells of the trichomes in the species from the genera *Dendrobium* (Davies and Turner, 2004), *Cyanaeorchis* (Pansarin and Maciel, 2017), *Polystachya* (Davies *et al.*, 2002) and *Maxillaria* (Davies, 2009). Lipid droplets were recorded in the edible trichomes of *Cyanaeorchis* (Pansarin and Maciel, 2017). Thus, the edible trichomes of orchids are more diverse in the types of food material compared with *Pinguicula*.

From a phylogenetic perspective, edible trichomes are symplesiomorphic for the *Pinguicula* species and are found in the species of the ‘starch’ grade (Fig. 4), and therefore this does not support a monophyletic group such as a synapomorphy. However, the edible trichomes are derived and are possibly a specialization for fly and bee pollinators that act as a food reward for these visitors.

Field observations are needed to answer the question of whether insects consume ‘starch’ trichomes of *Pinguicula* flowers and thus whether these structures can be regarded as pollinators’ rewards. Checking if there is a correlation between the amount of nectar produced and the number of trichomes with starch also seems interesting.

### Conclusion

Floral non-glandular trichomes play the role of edible trichomes in some *Pinguicula* species (*P. agnata*, *P. albida*, *P. ibarrae*, *P. martinezii*, *P. filifolia* and *P. gigantea*), which are primarily classified as bee-pollinated species that originated from Central and South America. It seems that in *Pinguicula* that are pollinated by other pollinator groups (Lepidoptera and hummingbirds), the non-glandular trichomes in the flowers play a role other than being a floral reward for their pollinators. However, even with a phylogenetic perspective, the gaps in knowledge are wide for several species, which does not permit a robust hypothesis. Thus, only when field studies have been undertaken can we be absolutely certain of the role of these trichomes.

## SUPPLEMENTARY DATA

Supplementary data are available online at https://academic.oup.com/aob and consist of Table S1: micromorphology and histochemistry analyses of the food material content in various type of the *Pinguicula* flower non-glandular trichomes.

mcaa123_suppl_Supplementary_Table_S1
