# Supplementary material for: Do food trichomes occur in Pinguicula (Lentibulariaceae) flowers?
Source: Ann Bot. 2020 Jun 27;126(6):1039–48. doi: 10.1093/aob/mcaa123 (PMC7596368; doi:10.1093/aob/mcaa123)
Supplement: mcaa123_suppl_Supplementary_Table_S1 [file mcaa123_suppl_supplementary_table_s1.docx]

**Table S1:** Micromorphology and histochemistry analyses of the food material content (starch grains, proteins bodies and lipid droplets) in various type of the *Pinguicula* flower non-glandular trichomes.

| **Species** | **Localization** | **Trichome morphology** | | | **Presence of starch grains** | **Presence of proteins body** | **Presence of lipid droplets** |
| --- | --- | --- | --- | --- | --- | --- | --- |
|  |  | **Structure** | **Cuticle surface** | **Number of cells** |  |  |  |
| *P. moctezumae* | Palate (entrance to the throat)/Throat | Multicellular uniseriate slender trichome. Rarely apical part can creat two branches, which consist of 2-3 cells. | Mostly cuticular striations are present on cuticle surface of all cells, rarely first basal cell contain smooth cuticle surface. | 3-9 | - | - | - |
|  | Basal part of the spur | Multicellular uniseriate slender trichome pointed by a single acute apical cell. | Cuticular striations are present on cuticle surface of all cells. | 2-5 | - | - | - |
| *P. rectifolia* | Palate/Throat/Basal part of the spur | Multicellular uniseriate slender trichome pointed by acute apical cell. | Cuticular striations are present on cuticle surface of all cells. | 4-9 | - | - | - |
| *P. moranensis* | Throat/Basal part of the spur | Multicellular uniseriate slender trichome pointed by a single acute to obtuse apical cell. | Cuticular striations are present on cuticle surface of all cells. | 3-10 | - | - | - |
| *P. emarginata* | Throat/Basal part of the spur | Multicellular uniseriate slender trichome pointed by a single acute apical cell. | Mostly cuticular striations are present on cuticle surface of all cells. Rarely first basal cell contain (almost) smooth cuticle surface or only apical cell contain cuticle striations, while rest of cells have (almost) smooth cuticle surface. | 3-6 | - | - | - |
| *P. esseriana* | Yellow spot (at the entrance to the throat) | Multicellular uniseriate slender clavate trichome. Apical part is pointed and consist of compact cells, while stalk consist of extended cells. | Cuticular striations are present on cuticle surface of all cells. | 6-11 | - | - | - |
|  | Throat/Basal part of the spur | Multicellular uniseriate thick trichome. Apical part of the trichome is pointed and consist of compact cells (rarely cells may have one cell outgrowth), whereas stalk consist of extended cells. | Cuticular striations are present on cuticle surface of all cells, except first basal cell, which have smooth cuticle surface. | 6-10 | - | - | - |
| *P. hemiepiphytica* | Palate/Throat/Basal part of the spur | Multicellular uniseriate long and slender trichome pointed by a single acute apical cell. | Cuticular striations are present on cuticle surface of all cells, except first basal cell, which have slight striations on cuticle surface. | 5-12 | - | - | - |
| *P. mesophytica* | Palate | Multicellular uniseriate long and slender trichome pointed by a single acute to obtuse apical cell. | All cells have smooth cuticle surface. | 4-13 | - | - | - |
|  | Throat/Basal part of the spur | Multicellular uniseriate slender trichome pointed by a single acute apical cell. | Mostly cuticular striations are present on cuticle surface of all cells, rarely single-cell head have smooth or almost smooth cuticular striations. | 3-7 | - | - | - |
| *P. agnata* | Entrance to the throat | Multicellular uniseriate slender clavate trichome. Head consist of few compact cells, while stalk consist of many extended cells. | Cuticular striations are present on cuticle surface of all cells, except first basal cell, which have slight striations or almost smooth cuticle surface. | 7-11 | + | - | - |
|  | Throat (in front) | Multicellular thick uniseriate compact trichome. | First basal cell (rarely second basal cell) have smooth cuticular surface, while rest of trichome cells have cuticular striations on cuticle surface. | 4-7 | + | - | - |
|  | Throat (at the entrance to the spur) | Multicellular (rounded cells) uniseriate thick compact trichome. Single-cell head may be rounded or pointed. | Apical cells (1-2) have almost smooth cuticle surface, while rest of trichome cells have smooth cuticle surface. | 3-8 | + | - | - |
|  | Throat (at the entrance to the spur) | Multicellular (extended cells) uniseriate slender trichome pointed by a acute single-cell head. | Apical cells (2-3) have cuticular striations or have almost smooth cuticle surface, whereas rest of trichome cells have smooth cuticle surface. | 3-7 | + | - | - |
| *P. gigantea* | Entrance to the throat | Multicellular uniseriate slender clavate trichome. Head consist of few compact cells, while stalk consist of many extended cells. | Cuticular striations are present on cuticle surface of all cells. | 9-16 | + | - | - |
|  | Throat (in front) | Multicellular uniseriate (rarely multiseriate) thick compact trichome. | First basal cell (rarely second basal cell) have smooth cuticular surface, while rest of trichome cells have cuticular striations on cuticle surface. | 4-8 | + | - | - |
|  | Throat (at the entrance to the spur) | Multicellular uniseriate slender trichome pointed by a single acute apical cell. | Basal cells (1-2) have smooth cuticular surface, while rest of trichome cells have cuticular striations on cuticle surface. | 4-8 | + | - | - |
| *P. ibarrae* | Entrance to the throat | Multicellular clavate slender trichome. Head is multiseriate and consist of few compact cells, while stalk is uniseriate and consist of many extended cells. | Basal cells (1-3) have smooth cuticular surface, whereas rest of trichome cells have cuticular striations on cuticle surface. | 6-12 | + | - | - |
|  | Throat (in front) | Multicellular uniseriate (rarely multiseriate) thick compact trichome. | First basal cell have smooth cuticular surface, while rest of trichome cells have cuticular striations on cuticle surface. | 4-8 | + | - | - |
|  | Throat (in front) | Multicellular uniseriate slender trichome. Apical part is rounded and consist of few compact head cells, while stalk consist of extended cells. | basal cells (1-2) have smooth cuticular surface, while rest of trichome cells have cuticular striations on cuticle surface. | 6-8 | + | - | - |
|  | Throat (at the entrance to the spur) | Multicellular uniseriate slender trichome pointed by a acute single-cell head. | In case longer trichomes (6-8 cells), first two basal cells have smooth cuticle surface, while rest trichome cells have striations. When trichomes are short (3-5 cells), only 1-2 apical cells have cuticular striations on cuticle surface. | 3-8 | + | - | - |
| *P. martinezii* | Entrance to the throat | Many-celled multiseriate slender clavate trichome. Apical part cells may contains one-celled outgrowths. | basal cells (1-2) have smooth cuticular surface, while rest of trichome cells have cuticular striations on cuticle surface. | 10-16 | + | - | - |
|  | Throat (in front) | Many-celled multiseriate slender clavate trichome. Apical part cells contains numerous one-celled outgrowths. | basal cells (1-2) have smooth cuticular surface, while rest of trichome cells have cuticular striations on cuticle surface. | 8-14 | + | - | - |
|  | Throat (at the entrance to the spur) | Multicellular uniseriate slender trichome pointed by a acute single-cell head. | In case longer trichomes (4-6 cells), 1-3 apical cells have cuticular striations on cuticle surface, whereas rest of trichome cells have smooth cuticle surface. When trichomes are short (2-3 cells), cuticular striations is various, there might be trichomes with smooth cuticle surface or with cuticular striations. | 2-6 | + | - | - |
| *P. albida* | Entrance to the throat | Multicellular clavate slender trichome. Head is mostly uniseriate (rarely multiseriate) and consist of compact cells, while stalk consist of extended cells. | Cuticular striations are present on cuticle surface of all cells, however first basal cell have slight cuticular striations. | 4-7 | + | - | - |
|  | Throat (in front) | Many-celled uniseriate or multiseriate thick compact trichome. | basal cells (1-2) have smooth cuticular surface, while rest of trichome cells have cuticular striations on cuticle surface. | 4-7 | + | - | - |
|  | Throat (at the entrance to the spur) | Multicellular uniseriate slender trichome pointed by a acute or obtuse apical cell. | Cuticular striations are present on cuticle surface of all cells. | 2-5 | + | - | - |
| *P. lusitanica* | Entrance to the throat | Multicellular clavate slender trichome. Many-celled head is mostly uniseriate (rarely multiseriate) and consist of compact cells, whereas stalk consist of extended cells. | Cuticular striations are present on cuticle surface of all cells. Head cells have distinct cuticle striations, while stalk cells have slight cuticular striations. | 6-10 | - | - | - |
|  | Entrance to the throat | Multicellular uniseriate short slender trichome. Single-cell head is rounded and set on thin stalk, which mostly consist of 2 cells. | Cuticular striations are present on cuticle surface of all cells. | 2-4 | - | - | - |
|  | Throat (in front and at the entrance to the spur) | Many-celled multiseriate thick compact trichome. | Apical cells contain cuticular striations on cuticle surface, while basal cells have mostly slight cuticular striations or smooth cuticle surface. | 5-7 | - | - | - |
| *P. vulgaris* subsp. *vulgaris* | Entrance to the throat, Throat (including entrance to the spur) | Multicellular clavate slender trichome. Many-celled head is mostly multiseriate (rarely uniseriate) and consist of compact cells, whereas stalk consist of extended cells. | Cuticular striations are present on cuticle surface of all cells (sometimes basal cell of stalk have slight cuticular striations or almost smooth cuticular surface). | 10-18 | - | - | - |
| *P. vulgaris* subsp*. bicolor* | Entrance to the throat, Throat (including entrance to the spur) | Multicellular clavate slender trichome. Many-celled head is mostly multiseriate (rarely uniseriate) and consist of compact cells, whereas stalk consist of extended cells. | Cuticular striations are present on cuticle surface of all cells (sometimes basal cell of stalk have slight cuticular striations or almost smooth cuticular surface). | 7-16 | - | - | - |
| *P. alpina* | Entrance to the throat, Throat (including entrance to the spur) | Multicelular uniseriate slender trichome. Apical part consist of compact cells, while basal part consist of extended cells (rarely apical part is pointed by a rounded 2-celled head) | Cuticular striations are present on cuticle surface of all cells. | 5-9 | - | - | - |
| *P. filifolia* | Entrance to the throat, Throat (in front) | Multicellular clavate mostly uniseriate long-stalked trichome. Apical part consist of compact cells, while basal part consist of extended cells (rarely apical part is pointed by 2-3-celled multiseriate head) | Cuticular striations are present on cuticle surface of all cells (1-2 basal cells have slight cuticular striations) | 7-8 | Not examined | Not examined | Not examined |
|  | Throat (including entrance to the spur) | Multicellular compact multiseriate short-stalked trichome. Rarely trichome might creat two many-celled branches. | Cuticular striations are present on cuticle surface of all cells, except first basal cell, which have slight striations on cuticle surface. | 7-10 | + | - | - |
